# An integrative analysis of UFMylation-related genes reveals a prognostic signature and identifies GDF7 as a novel therapeutic target in gastric cancer

**DOI:** 10.3389/fonc.2026.1853513

**Published:** 2026-07-27

**Authors:** Guohao Chen, Xian Gao, Lingchen Dai, Shukang Deng, Haoyang Wang, Xinkun Huang, Ying Feng

**Affiliations:** 1 Department of Gastrointestinal Surgery, Affiliated Hospital of Nantong University, Medical School of Nantong University, Nantong, Jiangsu, China; 2 Research Center of Clinical Medicine, Affiliated Hospital of Nantong University, Nantong, Jiangsu, China; 3 Department of Special Clinic, Changhai Hospital, Second Military Medical University, Shanghai, China; 4 Department of General Surgery, Nantong Tumor Hospital and Affiliated Tumor Hospital of Nantong University, Nantong, Jiangsu, China; 5 Nantong Key Laboratory of Gastrointestinal Oncology, Nantong, Jiangsu, China

**Keywords:** gastric cancer, GDF7, immune infiltration, signature, targeted therapy, UFMylation

## Abstract

**Background:**

Recent studies have shown that UFMylation plays an important role in cancer, but its specific function in gastric cancer (GC) remains to be fully elucidated. This study aimed to develop a prognostic signature based on UFMylation-related genes (URGs) for survival prediction in GC.

**Materials and methods:**

We screened for differentially expressed genes (DEGs) between GC and normal control samples using The Cancer Genome Atlas (TCGA) database. Based on eight core URGs, unsupervised consensus clustering (CC) was utilized to classify GC samples into distinct molecular subtypes. Key co-expression modules and functional pathways were identified via Weighted Gene Co-expression Network Analysis (WGCNA) and enrichment analyses. A prognostic model was then constructed by Least Absolute Shrinkage and Selection Operator (LASSO) Cox regression analysis, followed by independent validation in the GSE84437 dataset. The biological effects of growth differentiation factor 7 (GDF7) on GC progression were evaluated through both *in vitro* and *in vivo* experiments.

**Results:**

A prognostic risk model was successfully constructed using 13 signature genes: PGR, BVES, CAVIN2, CDH6, COL10A1, GDF7, WDR17, MMP16, NSG2, NPTX1, ZNF662, APOD, and LBH. Using this model, patients were stratified into high- and low-risk groups. The model displayed robust predictive performance and was identified as an independent prognostic factor. Immune infiltration analysis revealed significantly altered immune cell distributions between the two risk groups, and drug sensitivity assessments identified potential therapeutic agents tailored to each group. Functional experiments confirmed GDF7 acts as a critical regulator of the proliferation, invasion, and migration of GC cells.

**Conclusion:**

A novel GC prognostic model was created, and GDF7 was determined as a potential therapeutic target. This study offers insights into the GC molecular mechanism and contributes to the formulation of personalized treatment strategies.

## 1 Introduction

According to the GLOBOCAN 2022 data, gastric cancer (GC) ranks as the fifth leading malignancy in both incidence and mortality worldwide (1). Due to insidious early symptoms, the majority of patients are diagnosed at advanced stages, resulting in an unfavorable prognosis with a 5-year survival rate of less than 35% (1, 2). Currently, surgical resection combined with chemoradiotherapy remains the primary treatment modality; however, its clinical efficacy is heavily restricted by postoperative recurrence and drug resistance (2, 3). Although the recent emergence of targeted therapy and immunotherapy has brought hope to select patient cohorts, the overall prognosis remains unsatisfactory (4, 5).Therefore, delving into the underlying molecular mechanisms of GC to identify reliable prognostic biomarkers and therapeutic targets is of great clinical significance.

Post-translational modifications are key contributors to proteomic complexity and are crucial for modulating protein homeostasis (6, 7). Ubiquitin-like modification, known as UFMylation, is a newly identified post-translational modification with significant implications for cellular regulation and disease (8, 9). Dysregulated UFMylation has been demonstrated to be a critical factor in multiple aspects of cancer progression, including cell cycle regulation, proliferation, migration, and invasion (10–15). Research suggests that UFMylation exerts anti-tumor effects by counteracting p53 ubiquitination, thereby maintaining p53 stability (16). Conversely, UFMylation can enhance the transcriptional activity and stability of estrogen receptor, thereby promoting breast cancer (BC) progression (17). Furthermore, UFMylation is involved in modulating the tumor microenvironment (TME) and immune responses, suggesting that it serves as a potential target in treatment (7, 15). Additionally, the interaction of UFMylation with other post-translational modifications (e.g., SUMOylation, ubiquitination) increases complexity in regulation (14, 18). However, research on UFMylation in GC is still in its infancy, and its specific regulatory mechanisms and clinical relevance remain unclear. Thus, systematically deciphering UFMylation expression patterns in GC and evaluating their impact on prognosis holds promise for laying a new theoretical basis for precise GC treatment.

Using GC data in the Gene Expression Omnibus (GEO) and The Cancer Genome Atlas (TCGA), this study employed bioinformatic analyses to screen for key UFMylation-related genes (URGs). A prognostic model comprising 13 genes was constructed by LASSO regression, which demonstrated robust predictive capacity in the training and independent validation sets. Patients were assigned by this model into two risk groups (high and low). Further analyses revealed that the model was not only closely associated with patients’ clinicopathological data but also reflected immune infiltration status and predicted patient sensitivity to various chemotherapeutic agents. Moreover, *in vivo* and *in vitro* experiments were performed to validate the core gene, growth differentiation factor 7 (GDF7), in the proliferation, migration, and invasion of GC cells. The findings are expected to provide a potential target in GC treatment.

## 2 Materials and methods

### 2.1 Acquisition of the raw data

We acquired the gene expression and clinicopathological data of GC patients in the TCGA from the UCSC (https://xena.ucsc.edu). Additionally, the GSE84437 dataset was retrieved from the GEO database (https://www.ncbi.nlm.nih.gov/geo/) to serve as a completely independent external validation set. This dataset comprises global mRNA expression matrices and fully matched clinical follow-up data of 433 GC patients from the Asian Cancer Research Group, which were profiled on the Affymetrix Human Genome U133 Plus 2.0 Array platform (GPL6947). Previous studies identified eight URGs (UFL1, UBA5, UFC1, DDRGK1, CDK5RAP3, UFSP1, UFSP2, and UFM1) for subsequent analyses (8, 13).

### 2.2 Differentially expressed genes

Using the R “limma” package (v3.64.3), we screened DEGs with |logFC| >1 and adj.P <0.05 in GC and control samples in the TCGA (19). Only the overlapping genes were included to enhance the reliability. The analysis of variance results were visualized by the R “ggplot2” package (v4.0.0).

### 2.3 Unsupervised consensus clustering

We used the “ConsensusClusterPlus” package (v1.72.0) (20) and a Pearson correlation distance-based agglomerative k-means clustering algorithm, performing 1000 resampling on 80% of data. The cumulative distribution function curve was generated for the optimal number of clusters.

### 2.4 WGCNA

The R “WGCNA” package (v1.73) was utilized (21). A clustering tree was created to eliminate outliers. Afterward, an adjacent matrix was created by Pearson correlation analyses on all genes. Furthermore, an appropriate soft threshold parameter β was selected to calculate the gene correlation. To achieve this, we performed a scale-free network distribution on the connections between genes in the network (β: a soft threshold parameter emphasizing strong gene correlations while penalizing weak ones). We then transferred the results to the adjacent matrix and subsequently to the topology overlap matrix (TOM).

To include genes with similar expression patterns into the same module, hierarchical clustering was performed using 1-TOM, with a minimum module size of 300 genes. In the principal component analysis, module eigengene as the principal component represented the gene expression within modules. All genes within the module were pooled into one expression profile. Finally, highly correlated modules were merged (cutoff: dissimilarity <0.3).

### 2.5 Identification and validation of key URGs

After the module of interest was identified, we intersect DEGs and genes listing in the module of interest to obtain potential key URGs. They underwent both GO (22) and KEGG (23) analyses by the “ClusterProfiler” package (v4.16.0).

### 2.6 Construction of the prognostic model

Prognosis-related URGs were identified by the univariate Cox regression. A prognostic model was created by LASSO Cox regression with the R “glmnet” package (v4.1.10). Independent variables comprised the URG expression, while OS and survival status in GC patients in the TCGA were the dependent variables. We calculated the risk scores according to the gene expression and corresponding regression coefficients, based on which patients were stratified into two risk groups (low and high). The R “pROC” package (v1.19.0.1) was used to draw the survival curves. The model accuracy was further tested by GSE84437 validation.

### 2.7 Assessment of predictive capacity

The patient survival in both groups was assessed by the Kaplan-Meier (K-M) analysis using the “survminer” (v0.5.1) and “survival” (v3.8.3) packages. Subsequently, ROC curves were generated with the “survivalROC” package (v1.0.3.1) to assess specificity and sensitivity. To further assess the model’s predictive capacity, the signature in the TCGA underwent univariate/multivariate Cox regression analyses. Additionally, different clinical characteristics (stage, age, sex, and T/N/M stage) were analyzed in relation to GC risk scores. Finally, a nomogram was generated based on clinical characteristics and signature. Moreover, discrepancies in predicted and actual values were evaluated by calibration curves.

### 2.8 Immune landscape

The proportion of 22 tumor-infiltrating lymphocytes in the TME was calculated by the CIBERSORT algorithm from the *IOBR* package (https://github.com/IOBR/IOBR).

### 2.9 Drug sensitivity assessment

The prognostic model was utilized to predict the IC50 value of chemotherapeutic agents in both groups and to determine the patient sensitivity. The “pRRophetic” package (https://github.com/paulgeeleher/pRRophetic) was applied. Drug sensitivity assessment aimed to ensure the effectiveness of the prognostic model and to infer whether these chemotherapeutic agents are meaningful for the treatment.

### 2.10 Cell culture

A panel of GC cell lines (HGC-27, AGS, MKN-45, MKN-28, and SNU-216) was obtained from GeneChem (Shanghai, China). They were incubated in RPMI-1640 with 10% FBS and 1% penicillin/streptomycin at 100:10:1 under 5% CO_2_ at 37 °C.

### 2.11 Protein extraction and Western blotting

Cell lysates were added with protease and phosphatase inhibitors to extract the total protein. Protein concentration was measured by the BCA assay, followed by polyacrylamide gel electrophoresis. After being transferred onto PVDF membranes, the proteins were incubated with the anti-GDF7 primary antibody (#DF4097, 1:1000, Affinity) at 4 °C for 12 h, using GAPDH (60004-1-Ig, 1:250,000, Proteintech) as an internal reference. The membranes were then incubated with secondary antibodies (#A0216 or #A0208, 1:2500, Beyotime) for 1h and developed by ECL solution. The relative immunoblot bands are compared using the prestained protein marker (Vazyme Biotech Co., Ltd., MP102-01) (**Supplementary Figure 1**).

### 2.12 Cell transfection

Cell transfection was performed as previously described (24). To silence GDF7 expression, a GDF7 siRNA (si-GDF7) and a control siRNA (siNC) were chemically synthesized by GeneAdv (Suzhou, China). Cells cultured in 6-well plates were transfected with Lipofectamine 3000 (Invitrogen, USA) using GC-specific siNC. 48 h after transfection, cells were used for subsequent analyses.

### 2.13 EdU

EdU was performed in accordance with our previous study (25). DNA synthesis was assessed by Click-iT EdU Imaging Kit (Bristol-Myers, Beijing) in accordance with the manufacturer’s instructions.

### 2.14 Colony formation assay

After 14 days of incubation in a 6-well plate, 1000 cells were fixed with 4% paraformaldehyde for 30 min and subjected to 30 min of 1% crystal violet staining.

### 2.15 Transwell assay

The transfected cells (5×10^4^) were suspended in a 200 μL FBS-free medium. The upper chamber with or without Matrigel was then added with the cell suspension, while medium with 20% FBS was added to the lower chamber. 36 h later, the cells were fixed and stained, followed by PBS washing. The supernatant cells were carefully removed with cotton swabs. The cells were finally selected randomly and counted in the field of view under a microscope.

### 2.16 Wound healing assay

After 5×10^5^ cells were cultured in a 6-well plate until fusion, they were scratched by a 200 μL pipette tip and placed in basal medium. Under an inverted microscope, each wound was photographed at 0 h and 24 h after removal of detached cell debris by PBS. The migration distance was calculated by ImageJ.

### 2.17 *In vivo* peritoneal metastasis assay in nude mice

15 nude male mice (4 weeks old, the Model Animal Research Center of Nantong University, Nantong, China) were randomized into a si-GDF7 group and a control group (n=5 each). Each mouse was injected subcutaneously with 200 μL of cell suspension (2×10^6^ cells) via the right axilla. We observed the growth status regularly, and recorded the tumor weight and volume every three days. 23 d later, the nude mice were sacrificed, and the tumor tissues were stripped for weighing, photographing, and recording. The subcutaneous tumor growth curve was further drawn. The mice were euthanized with isoflurane anesthesia (induced concentration, 3–4%) for 2–3 min and maintained anesthetized using at 1–1.5% isoflurane, and the remaining mice were then sacrificed using CO_2_ (28% volume displacement per min) after the experiment was completed. Their remains were placed in white sealing bags and sent to the Nantong University Laboratory Animal Center for centralized and safe disposal. The Laboratory Animal Ethics Committee of Nantong University approved all animal experiments. Safe disposal. Ethics Approval Number: S20260130-008. All experiments were reported in accordance with the ARRIVE guidelines.

### 2.18 Data analysis

All experiments were repeated in triplicate. R software (v4.4.1) was utilized. Mann-Whitney U test for continuous data and chi-square test (or Fisher’s exact test for small sample sizes) for categorical data were conducted in the two groups. P<0.05 (*P<0.05, **P<0.01, and ***P<0.001) was deemed statistically significant.

## 3 Results

### 3.1 DEGs identified

**Figure 1:**
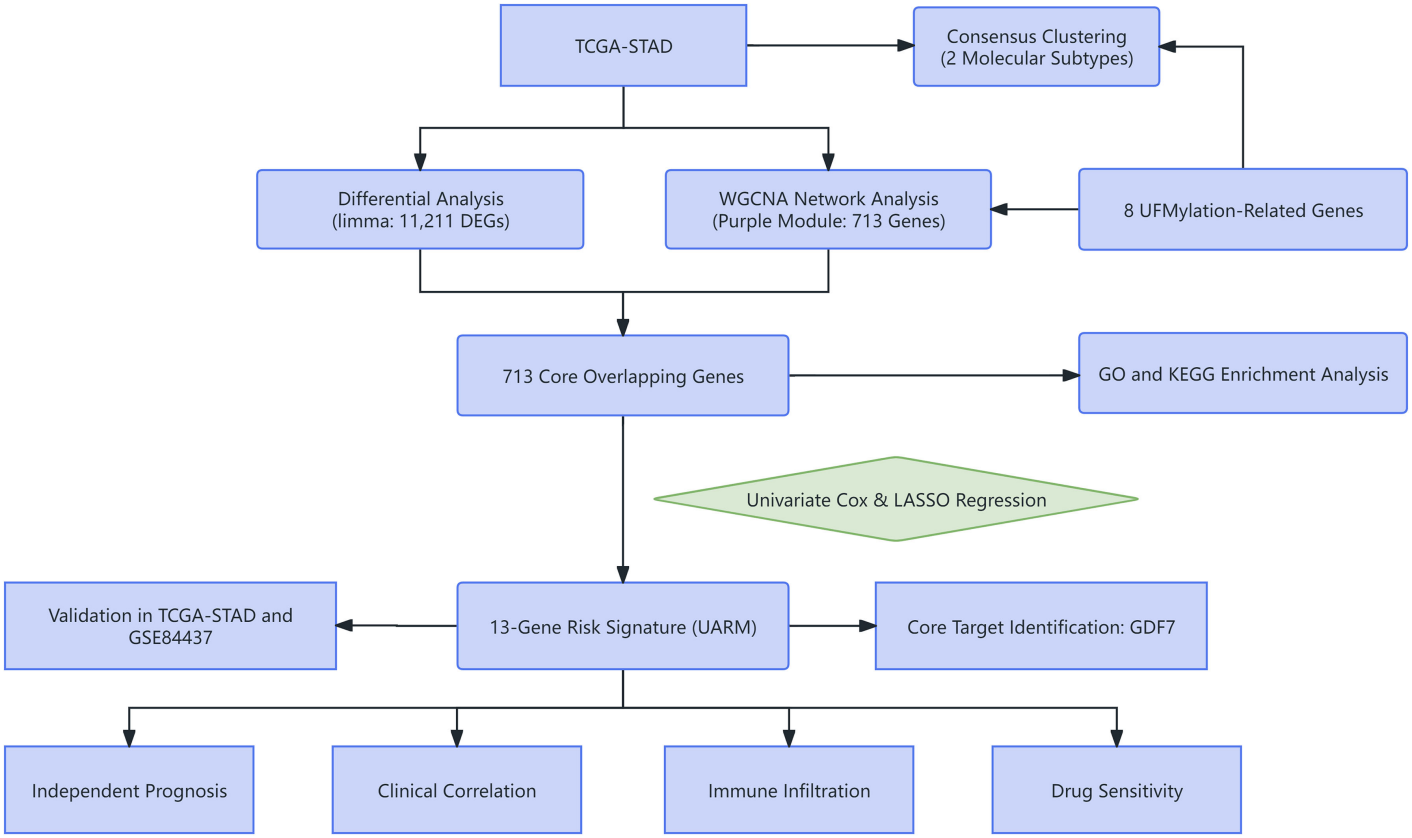
Flowchart of this study. We systematically screened and validated key UFMylation-related markers in gastric cancer. Differential expression analysis of TCGA-STAD identified 11,211 DEGs, and unsupervised consensus clustering of 8 core URGs revealed two molecular subtypes. WGCNA, univariate Cox, and LASSO regression refined overlapping candidates into a 13-gene prognostic risk model (UARM). Clinical survival and immune infiltration analyses demonstrated its stable predictability. GDF7 emerged as the pivotal prognostic target, and its oncogenic roles were validated *in vitro* and *in vivo*.

**Figure 2:**
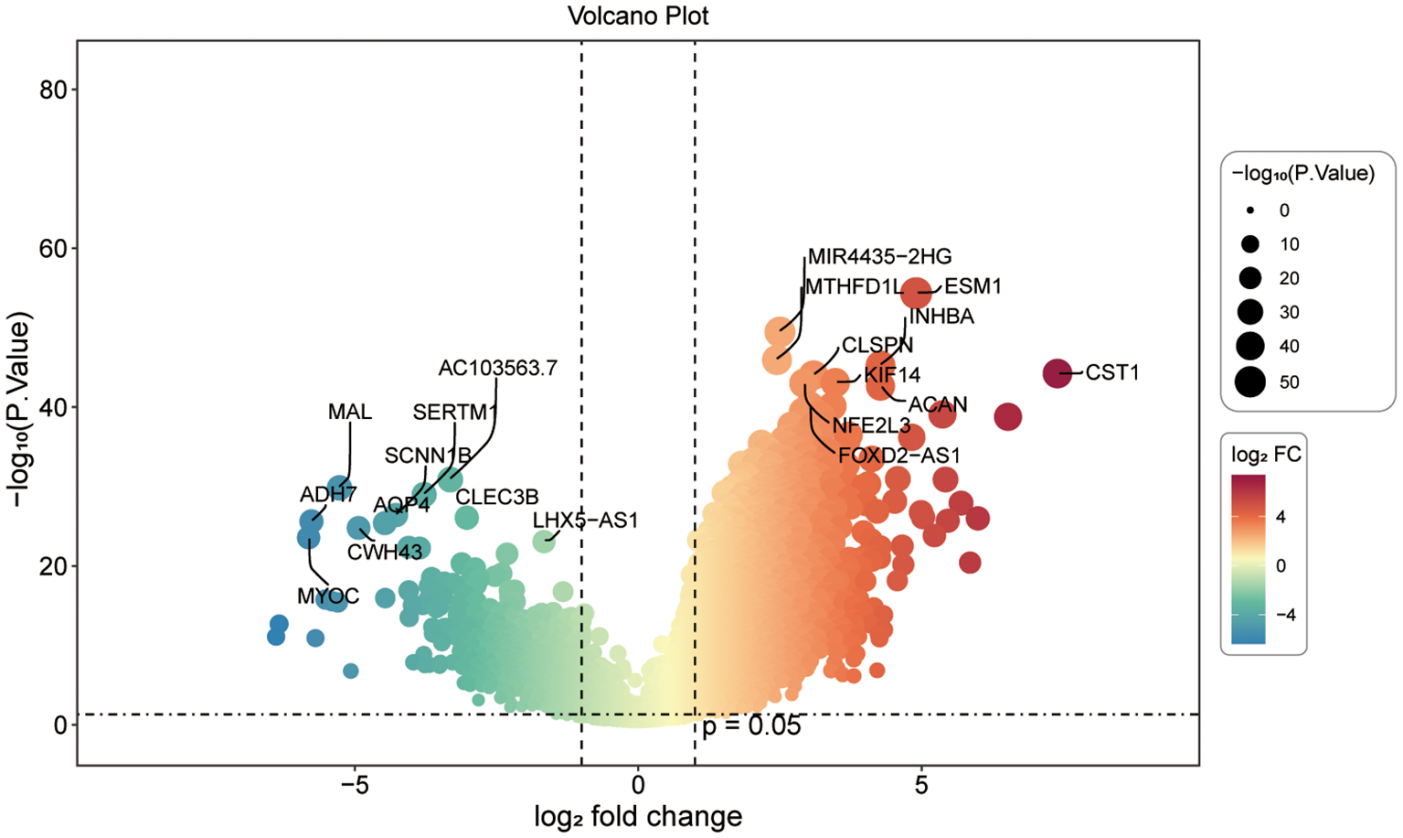
Identification of DEGs in the TCGA-STAD cohort. Red and blue dots represent statistically significant up- and downregulated genes, respectively, under rigorous filtration thresholds of an adjusted P-value <0.05 and an absolute log2 fold-change (|log2FC| >1).

The comprehensive study design and core target screening pipeline of this study are schematically illustrated in **Figure 1**. A total of 11,211 DEGs (9867 upregulated and 1344 downregulated) were identified between GC and normal samples. The gene expression in both groups was visualized by the volcano (**Figure 2**) plots.

### 3.2 Unsupervised CC

**Figure 3:**
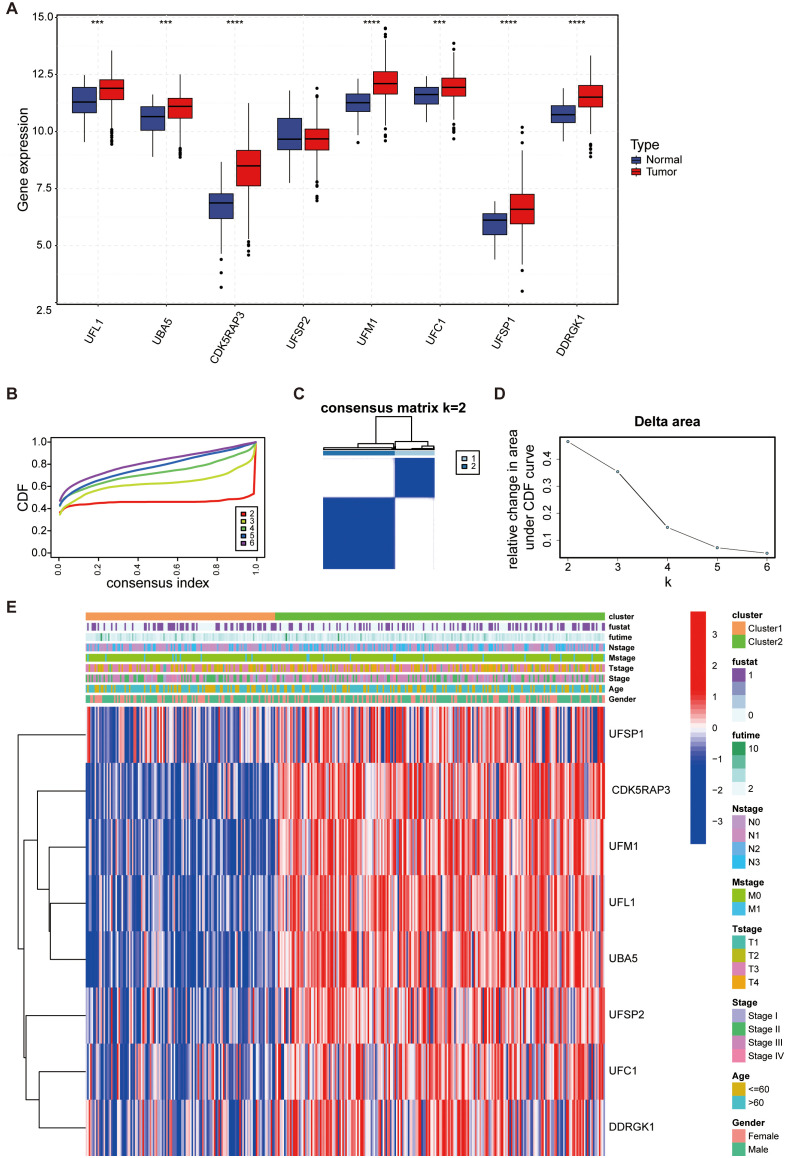
Expression landscape and unsupervised CC of the 8 core URGs in the TCGA-STAD cohort. **(A)** Boxplot comparing the transcriptomic expression levels of the 8 core URGs between GC tissues and normal control tissues. **(B)** Cumulative distribution function curves for CC across different cluster numbers (k=2-6). **(C)** Consensus matrix heatmap illustrating sharp and stable sample partitioning boundaries at k=2. **(D)** Delta area plot for the relative change in the area under the CDF curve across various k values. **(E)** Clustering heatmap for the distinct expression patterns of the 8 core URGs between Cluster 1 and Cluster 2.

The expression levels of 8 URGs were compared between GC tissues and normal tissues in the TCGA-STAD dataset (**Figure 3A**). The results showed that the expression levels of UFL1, UBA5, CDK5RAP3, UFM1, UFC1, UFSP1 and DDRGK1 were upregulated in cancer tissues, whereas the expression levels of UFSP2 exhibited no statistically significant alteration despite a slight downward trend (**Figure 3A**). Subsequently, unsupervised CC was used to classify the TCGA cohort into distinct clusters based on the 8 URGs. The number of clusters (k) was evaluated, and for each k value, a cumulative distribution function curve was generated (**Figures 3B–D**). The k value was determined to be 2, achieving greatest inter-cluster discrimination with least overlap. Patients were thus assigned into Cluster 1 (*n* = 127) and Cluster 2 (*n* = 221), and URGs had higher levels in Cluster 2 (**Figure 3E**).

### 3.3 WGCNA and enrichment analyses

**Figure 4:**
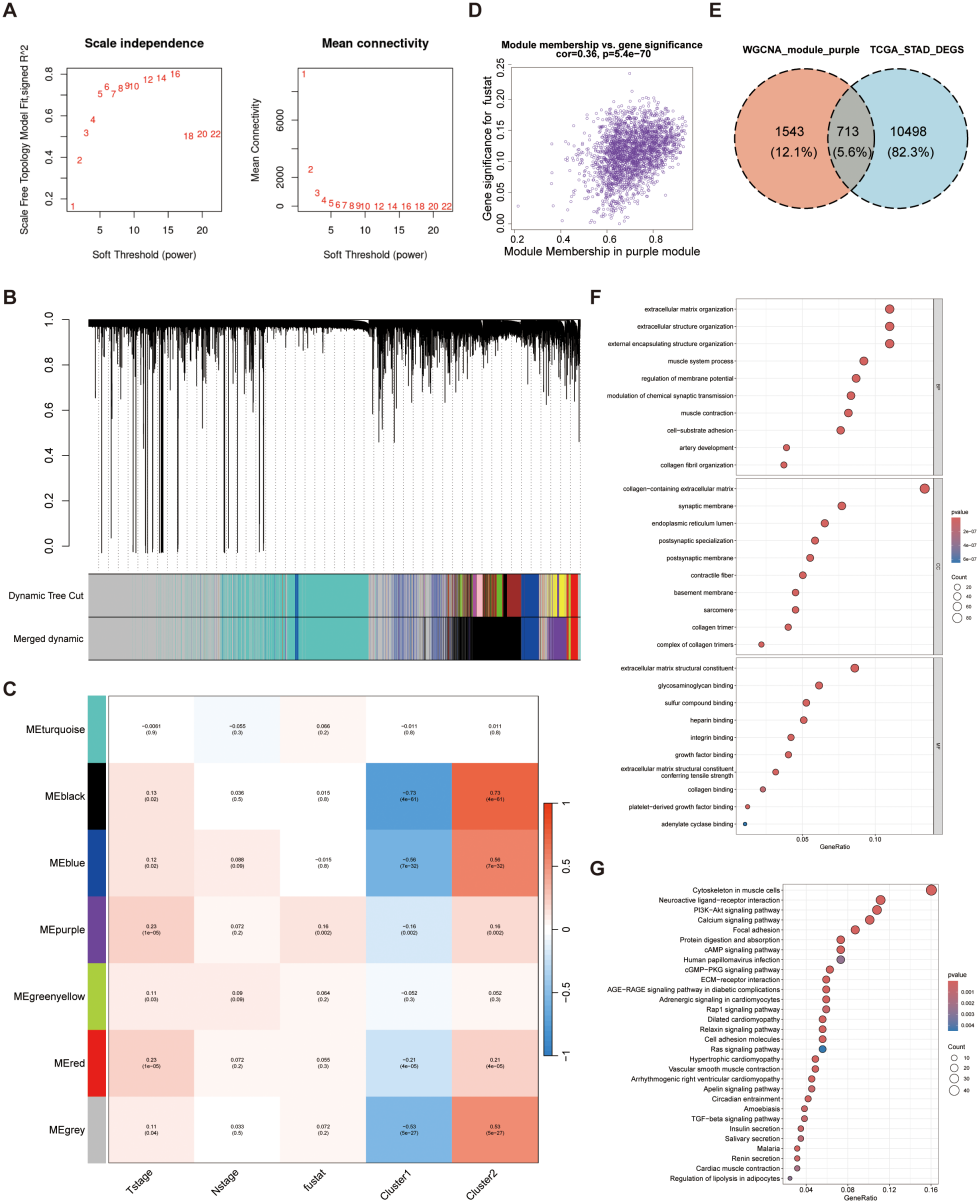
WGCNA and enrichment analyses. **(A)** Network topology analysis for determining the soft-thresholding power. **(B)** Merged dynamic tree of genes with dissimilarity based on topological overlap and module colors. **(C)** Correlation between different modules and T stage, N stage, clinical status and GC subtypes. **(D)** Scatter plot between module membership in purple module and the gene significance for GC. **(E)** Venn diagram for the intersections between DEGs and WGCNA. **(F)** GO functional enrichment of 713 intersection genes. **(G)** KEGG pathway enrichment of 713 intersection genes.

Network topology analysis was conducted to determine the soft power, and it was set to 16 in the WGCNA (**Figure 4A**). We created a hierarchical clustering tree, yielding seven modules (**Figure 4B**). By comparing the correlation of modules with clinical characteristics, the purple module showed the highest correlations with T stage, clinical status, and GC subtypes (**Figure 4C**). Therefore, the purple module served as the primary module. A significant positive correlation of MM with GS in this module was revealed by the correlation analysis (cor = 0.36, P<0.001, **Figure 4D**). Based on WGCNA and DEG data, a Venn diagram was constructed to identify intersections (**Figure 4E**).

These genes underwent GO and KEGG analyses for their potential functions. In the GO analysis, they were primarily enriched in muscle contraction, collagen fibril organization, artery development, and synaptic transmission modulation (biological process); basement membrane, collagen-containing extracellular matrix, sarcomere, and postsynaptic membrane (cellular component); extracellular matrix structural constituent, collagen binding, and integrin binding (molecular function) (**Figure 4F**). In the KEGG analysis, they were enriched primarily in cardiac muscle contraction, TGF-beta signaling, ECM-receptor interaction, insulin secretion, and PI3K-Akt signaling (**Figure 4G**).

### 3.4 Model evaluation and validation

**Figure 5:**
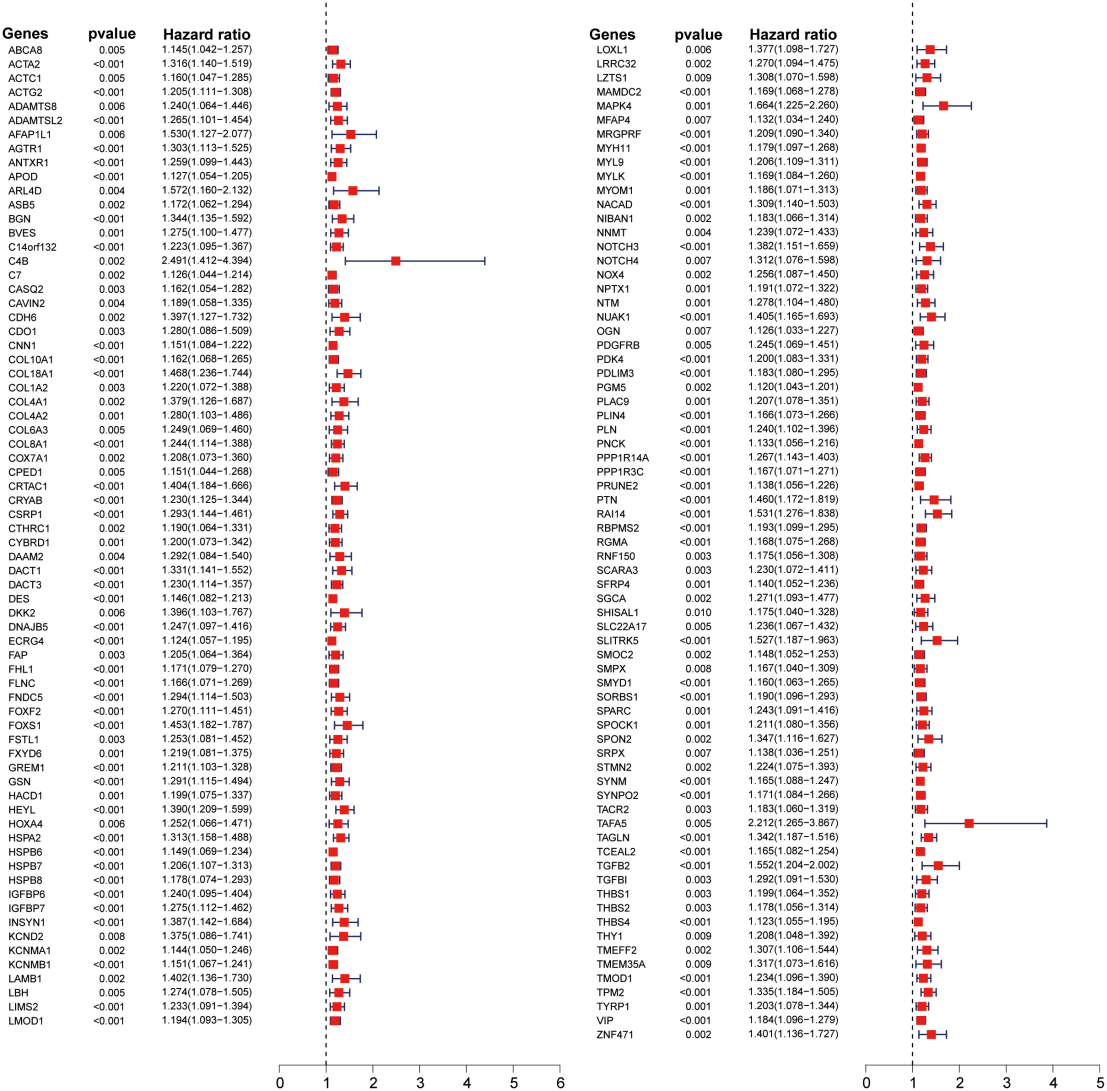
Screening of prognosis-associated intersection genes via univariate Cox regression. The forest plot shows hazard ratios (HRs) with 95% confidence intervals (CIs) for each gene. The x−axis (log scale) represents HR, and the y−axis lists gene symbols. The vertical dashed line indicates HR = 1. Error bars represent 95% CIs.

**Figure 6:**
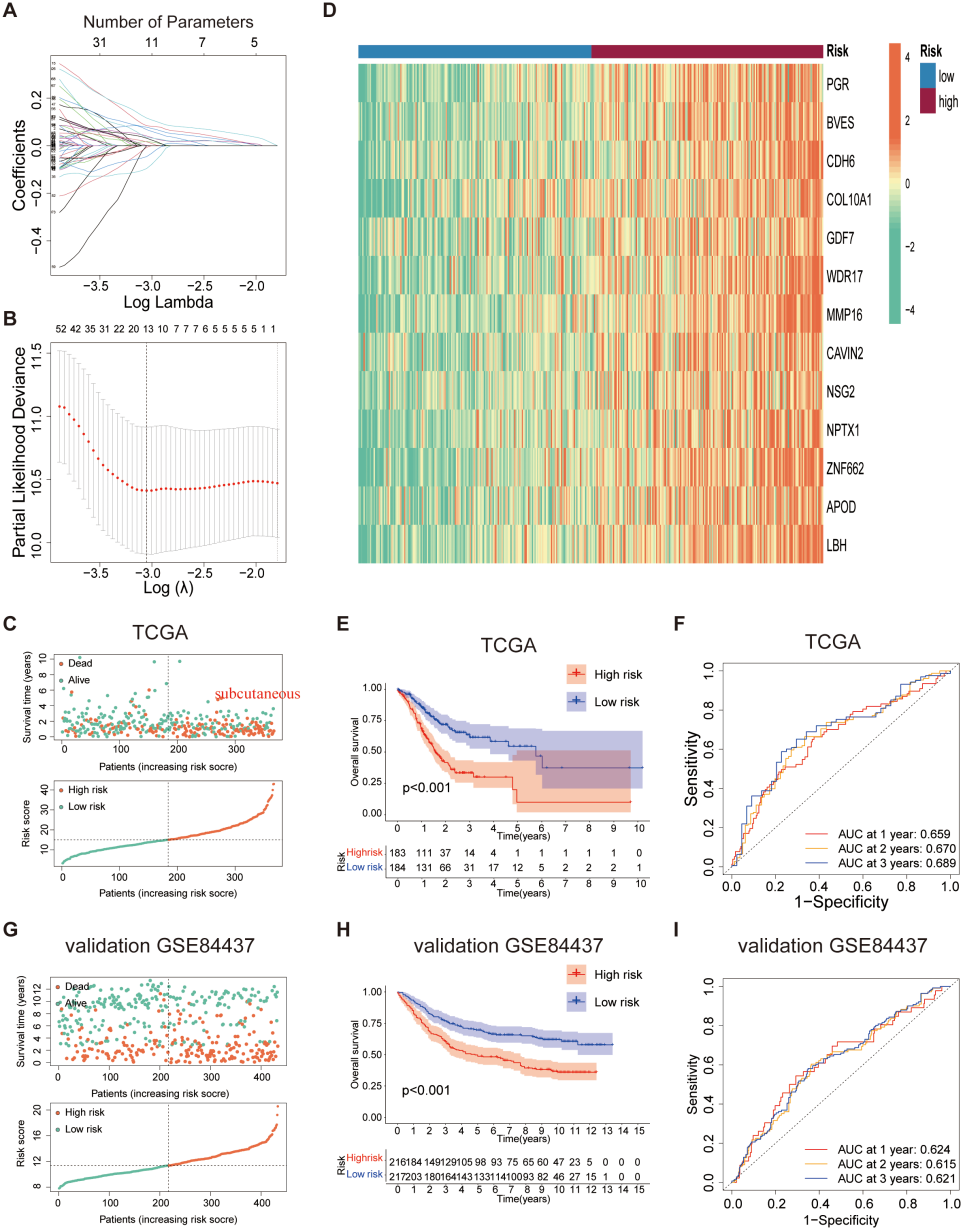
Evaluation and validation of prognostic model based on URGs. **(A)** LASSO coefficient curve. **(B)** Coefficient distribution chart from ten-fold cross-validation for the LASSO model, with λ as the tuning parameter. **(C, G)** Risk score distribution and survival status for GC patients in the low-risk and high-risk groups in TCGA cohort **(C)**, and GSE84437 cohort **(G)**. **(D)** The expression of the 13 model genes for GC patients in the low-risk and high-risk groups in TCGA cohort. **(E, F, K–M)** survival analysis in TCGA cohort **(E)**, and GSE84437 cohort **(H)**. **(F, I)** ROC analysis of the survival rates of patients in TCGA cohort **(F)**, and GSE84437 cohort **(I)**.

First, in the TCGA cohort, URGs were screened by univariate Cox regression from the previously identified intersection genes (**Figure 5**). Then LASSO Cox regression was conducted to further refine the selection of URGs by their predictive value (**Figures 6A, B**). Ultimately, 13 genes were selected. The URG-associated risk model (UARM) was created as follows: Risk score = -0.096 × PGR - 0.007 × BVES - 0.046 × CAVIN2 + 0.068 × CDH6 + 0.084 × COL10A1 + 0.018 × GDF7 + 0.078 × WDR17 + 0.041 × MMP16 + 0.001 × NSG2 + 0.031 × NPTX1 + 0.116 × ZNF662 + 0.016 × APOD + 0.035 × LBH.

By evaluating the gene expression, the prognostic risk in GC was determined in the TCGA cohort. The patients were then assigned into two risk groups. The high-risk group had higher mortality (**Figure 6C**). Moreover, the two groups had obvious differences in the prognostic gene expression (**Figure 6D**). Besides, the high-risk group had an obviously lower survival rate (P<0.001) (**Figure 6E**). To validate the prognostic model, 1-, 2-, and 3-year ROC curves were generated in the TCGA cohort, yielding AUCs of 0.659, 0.670, and 0.689, respectively (**Figure 6F**).

Furthermore, we validated the model accuracy in the GSE84437 validation set. This set reflected the survival status and characteristic gene expression in both groups in the TCGA cohort (**Figure 6G**). The K-M curves still indicated a significantly lower survival rate in the high-risk group (P<0.001) (**Figure 6H**). The AUCs were 0.624, 0.615, and 0.621 for 1-, 2-, and 3-year predictions, demonstrating the prognostic model’s excellent predictive capability (**Figure 6I**). These results highlight the model’s potential as a potent tool for GC prognostic prediction.

### 3.5 Validation of UARM

**Figure 7:**
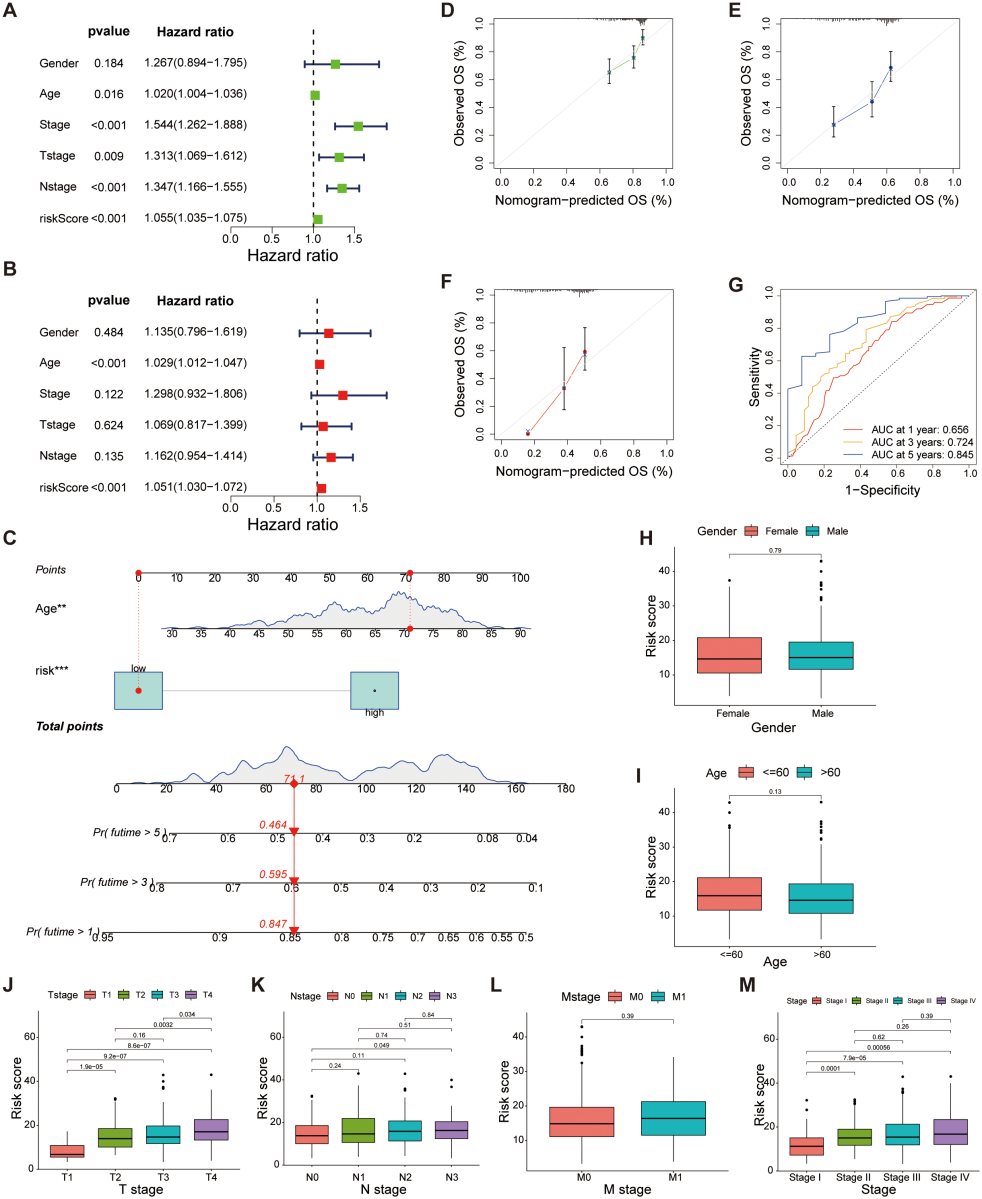
Validation of UARM as an independent prognostic factor. **(A)** Forest plot from univariate Cox regression analysis with combined clinical features and risk scores. **(B)** Forest plot from multivariate Cox regression analysis with integrated clinical features and risk scores. **(C)** Nomogram created based on clinical features and risk scores. **(D-F)** Calibration plots for 1- **(D)**, 3- **(E)**, and 5-year **(F)** predictions by the nomogram. **(G)** ROC curves for the assessment of clinical features and the risk score. **(H)** Correlation analysis of UARM and gender. **(I)** Correlation analysis of UARM and age. **(J)** Correlation analysis of UARM and T stage. **(K)** Correlation analysis of UARM and N stage. **(L)** Correlation analysis of UARM and M stage. **(M)** Correlation analysis of UARM and stage.

The model was determined as a prognostic factor for GC. The HR for UARM was 1.055 (95% CI 1.035-1.075) in the univariate regression analysis (**Figure 7A**), and it was 1.051 (95% CI 1.030-1.072) in the multivariate regression analysis (**Figure 7B**). Subsequently, a nomogram was created based on UARM for 1-, 2-, and 3-year survival prediction in GC (**Figure 7C**). It was assessed for predictive accuracy by calibration curves, and the predicted and observed outcomes were consistent (**Figures 7D–F**). Furthermore, the ROC curve demonstrated that this nomogram was effective in distinguishing outcomes, yielding AUCs of 0.656, 0.724, and 0.845 for 1-, 2-, and 3-year predictions (**Figure 7G**).

Additionally, the high-risk group was dominated by GC patients with higher T stage, N stage and TNM stage (**Figures 7H–M**). Conversely, GDF7 showed no relation with gender, age, or M stage (**Figures 7H–M**), underscoring its biological relevance. Thus, the risk score may be reliable as a prognostic factor for GC.

### 3.6 Immune cell infiltration

**Figure 8:**
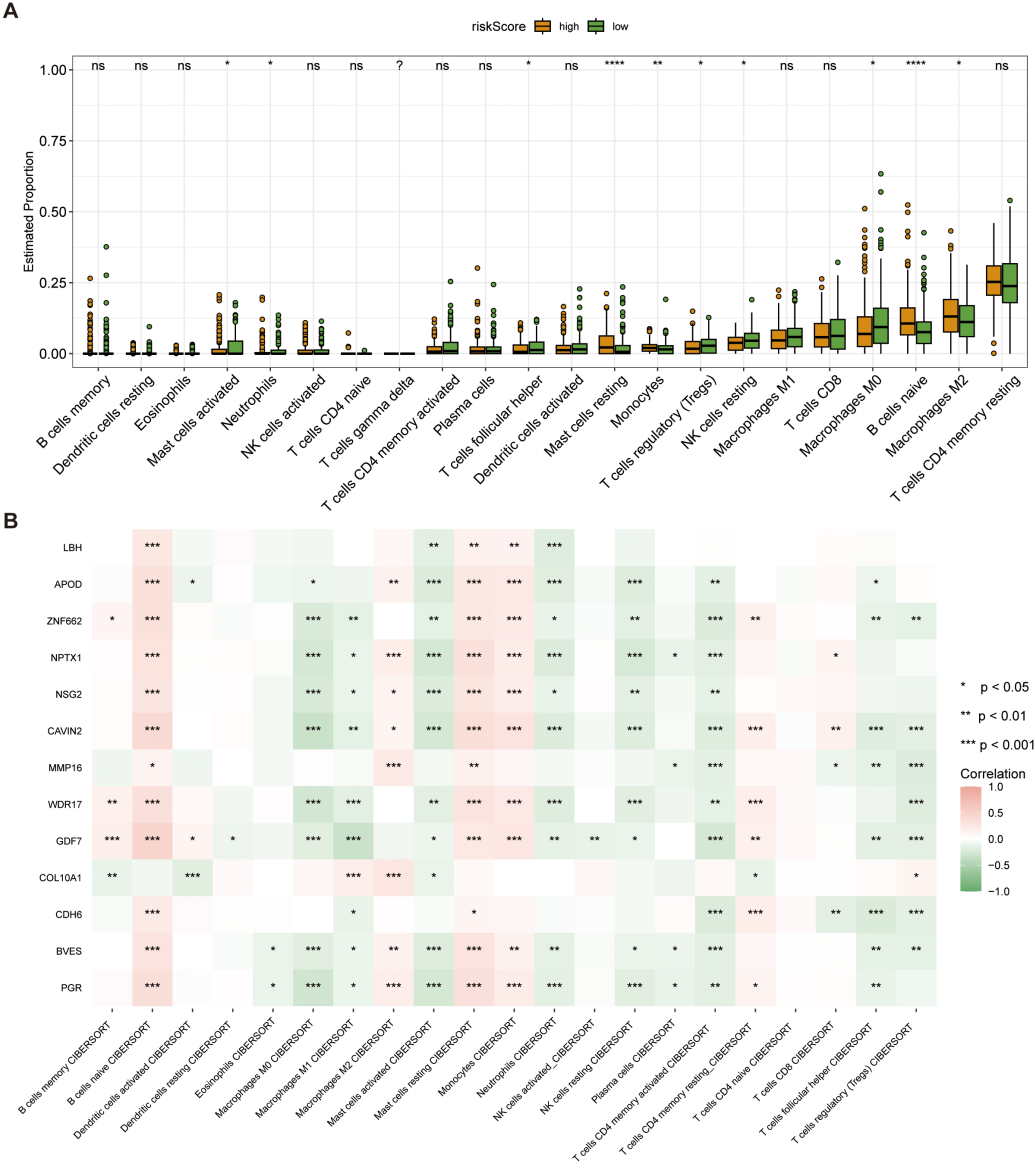
Immune factors in two risk groups. **(A)** Box plot illustrating the infiltration levels of different kinds of immune cells. **(B)** Correlation between 13 characteristic genes of UARM and immune cells.

By employing the CIBERSORT algorithm, we detected a notable increase in mast resting cells (P<0.0001), naive B cells (P<0.0001), monocytes (P<0.01), and M2 macrophages (P<0.05). Conversely, activated mast cells, neutrophils, follicular helper T cells, Tregs, resting NK cells (all P<0.05), and M0 macrophages relatively decreased (P<0.05) (**Figure 8A**). In accordance with the findings of the correlation analysis, PGR, BVES, CAVIN2, CDH6, COL10A1, GDF7, WDR17, MMP16, NSG2, NPTX1, ZNF662, APOD and LBH exhibited significant connection with a wide variety of immune cells (**Figure 8B**).

### 3.7 Correlations between UARM and drug sensitivity

**Figure 9:**
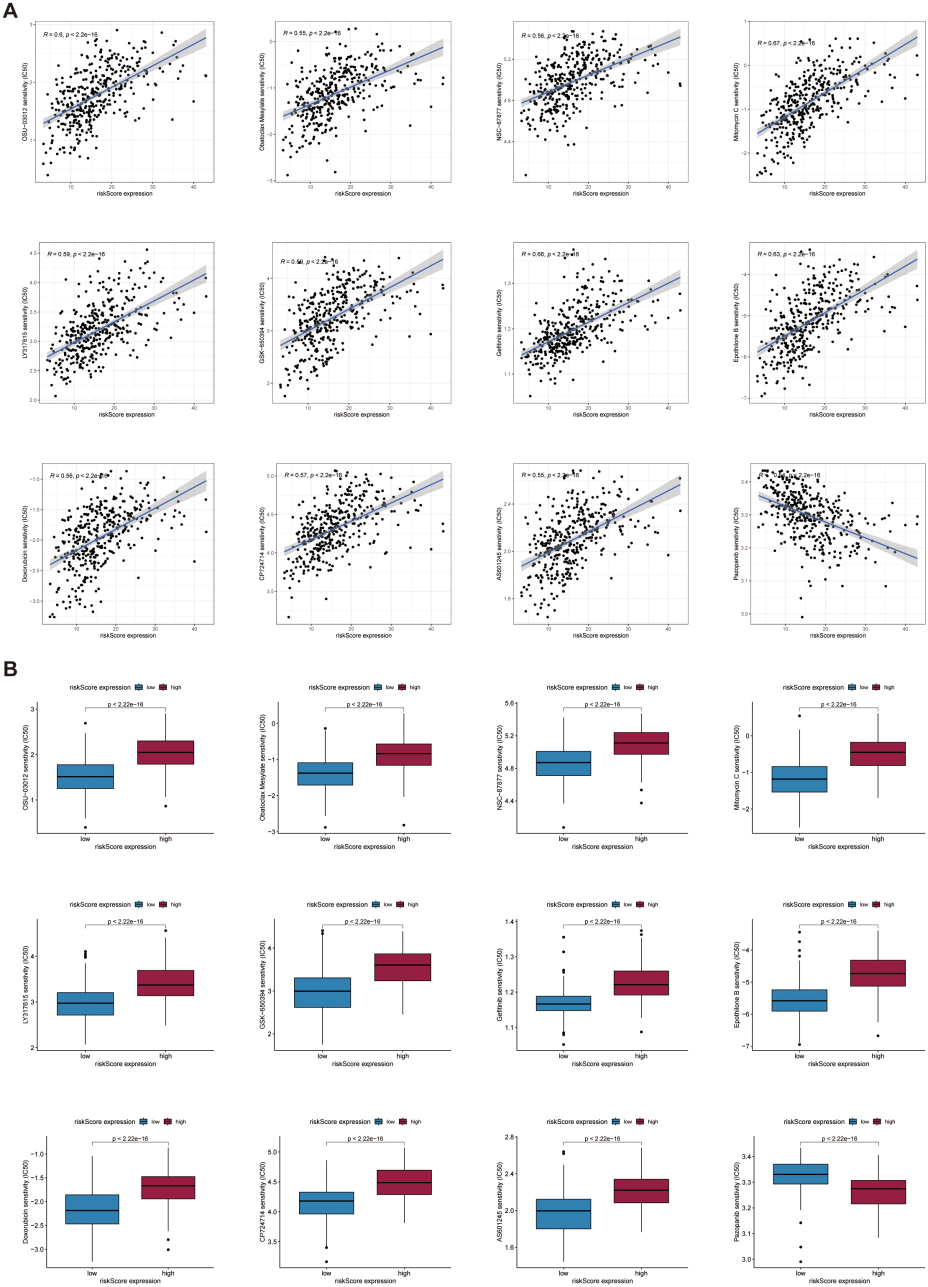
The relationship between the risk assessment model and common targeted drugs. **(A)** Sensitivity analysis of the top 12 drugs targeting UARM. **(B)** IC50 values of the 12 drugs in high- and low- risk group.

To enhance the precision of treatment strategies for GC, patient responses to various anticancer drugs in each group were evaluated. We screened 12 chemotherapeutic agents closely linked to RiskScore (|cor| > 0.5): OSU-03012, Obatoclax Mesylate, NSC-87877, Mitomycin C, LY317615, GSK-650394, Gefitinib, Epothilone B, Doxorubicin, CP724714, AS601245, and Pazopanib (**Figure 9A**). The high-risk group exhibited higher sensitivity to Pazopanib (**Figure 9B**), while the low-risk group displayed higher sensitivity to OSU-03012, Obatoclax Mesylate, NSC-87877, Mitomycin C, LY317615, GSK-650394, Gefitinib, Epothilone B, Doxorubicin, CP724714, and AS601245 (**Figure 9B**). Therefore, personalized medicine is important, in which the most effective targeted therapy or chemotherapy can be selected according to the risk status. Furthermore, understanding the response of each group to specific anticancer drugs can greatly contribute to GC treatment and outcomes.

### 3.8 GDF7 regulated GC cell proliferation, migration, and invasion

**Figure 10:**
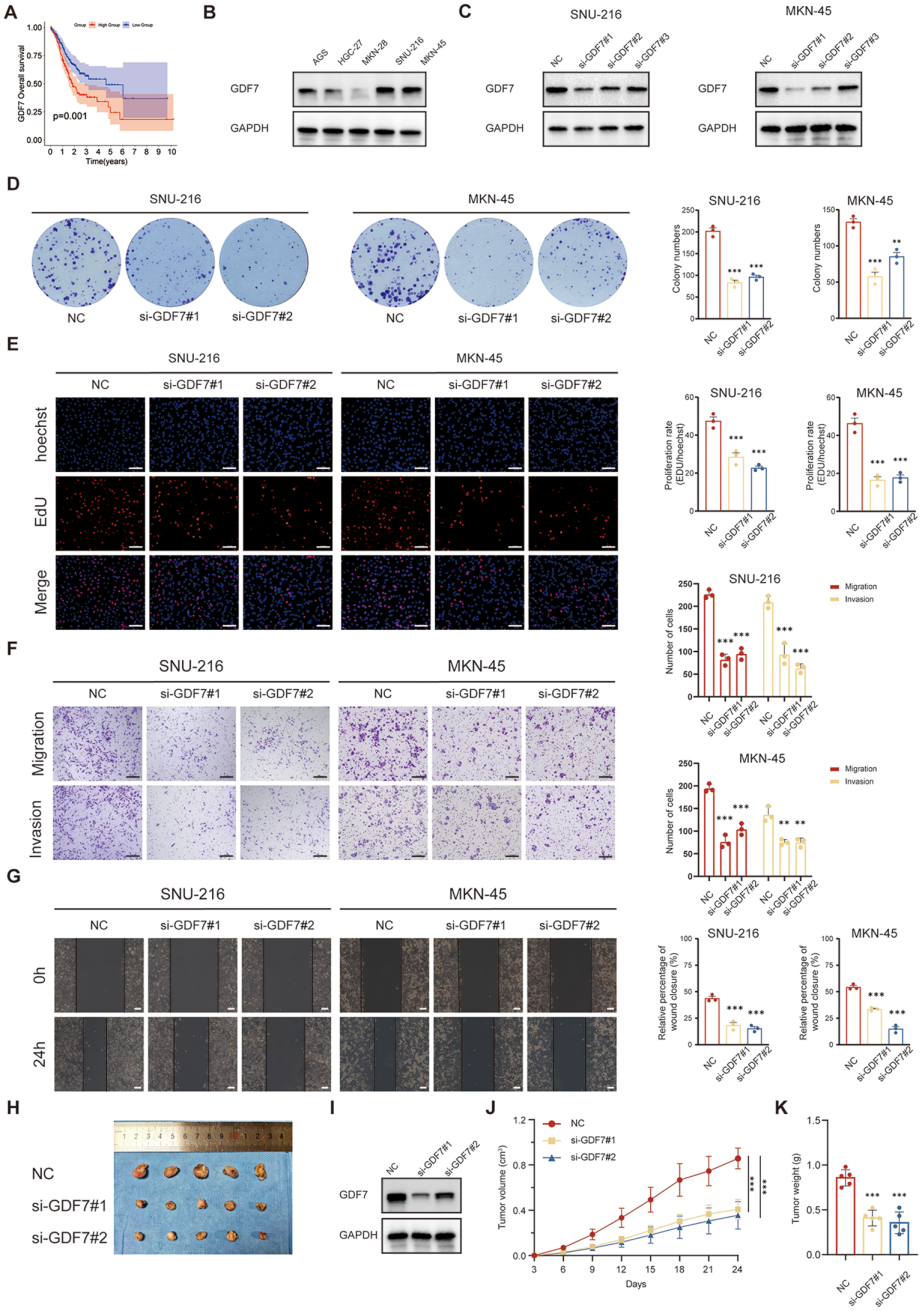
GDF7 regulates GC cell proliferation, migration and invasion. **(A)** K-M survival analysis displayed high expression of GDF7 correlated with poor prognosis in GC patients in TCGA cohort. **(B)** Western blotting for GDF7 expression in GC cell lines. **(C)** Western blotting for GDF7 levels in SNU-216 and MKN-45 cells transfected with control (NC) and siRNA. **(D, E)** Colony formation assay and EdU assay for the impact of si-GDF7 on cell proliferation, Scale bars = 100 µm. **(F, G)** Transwell and wound healing assays for the impact of si-GDF7 on migration of SNU-216 and MKN-45 cells, Scale bars = 200 µm **(F)**, Scale bars = 100 µm **(G)**. **(H)** Xenograft model in nude mice. **(I)** Western blotting analysis of the GDF7 protein expression levels within the harvested subcutaneous tumor tissues. **(J)** Tumor volume in the nude mice. **(K)** Tumor growth rate in the nude mice.

The selection of GDF7 for functional investigation was based on multiple considerations. High GDF7 expression was consistently associated with poor overall survival in our TCGA analysis (**Figure 10A**). Additionally, the role of GDF7 in GC remains completely unexplored, representing a novel candidate. Therefore, we investigated whether GDF7 acts as an oncogene or tumor suppressor in GC.

Initial analysis of GDF7 protein levels in GC cell lines revealed its most significant upregulation in SNU-216 and MKN-45 cells (**Figure 10B**). Western blotting results confirmed that GDF7 protein expression was knocked down by si-GDF7#1 and si-GDF7#2 in both GC cell lines (**Figure 10C**). Subsequently, colony formation and EdU assays demonstrated that GDF7 silencing significantly inhibited GC cell viability (**Figures 10D, E**). Wound healing and Transwell assays further demonstrated that GDF7 knockdown significantly suppressed GC cell migration and invasion (**Figures 10F, G**).

Finally, tumor growth was obviously delayed in mice injected with si-GDF7 MKN-45 cells in subcutaneous tumor models of nude mice (**Figure 10H**). Western blotting analysis of the subcutaneous tumor tissues further confirmed the stable *in vivo* knockdown efficiency of GDF7 protein (**Figure 10I**). Subcutaneous tumor volume and weight were significantly smaller in si-GDF7 cells (**Figures 10I, J**). To sum up, GDF7 silencing impairs proliferation, invasion, and migration of GC cells.

## 4 Discussion

GC is still the most frequent and lethal malignancy globally (1). Surgical resection is typically the treatment for early-stage GC, but chemotherapy is needed for patients in late stage, with a low 5-year survival rate (2, 5, 26). To obtain better GC outcomes, therefore, the efficacy of chemotherapy or targeted therapies should be improved (26). With recent advances in gene sequencing techniques, new methods have emerged for elucidating the molecular mechanism underlying GC onset and progression.

As a new ubiquitin-like modification, UFMylation is a significant player in tumor development and progression (8). UFM1, similar to ubiquitin, covalently binds to its substrate proteins via an enzyme reaction involving E1 (UFM1-activating enzyme), E2 (UFM1-conjugating enzyme 1), and E3 (UFM1-specific ligase 1) (8, 13). Research suggests that dysregulated UFMylation is a key factor in various diseases (9, 11, 17). Nonetheless, research elucidating the role of UFMylation in GC is still lacking, and URGs still have unclear relationships with GC prognosis. This study explored URGs and their potential biological functions, and created a GC prognostic model.

Based on eight URGs, unsupervised CC was used to classify the GC samples into two subtypes. Subsequently, we identified a key module (the purple module) significantly associated with both prognosis and subtypes via WGCNA, which contained 713 DEGs closely linked to cancer development. Finally, we constructed a novel 13-gene UARM by LASSO Cox regression, comprising PGR, BVES, CAVIN2, CDH6, COL10A1, GDF7, WDR17, MMP16, NSG2, NPTX1, ZNF662, APOD, and LBH.

This UARM demonstrated robust predictive capacity in the training and independent validation sets, by which patients were stratified into two risk groups. Furthermore, the immune infiltration analysis revealed a significant increase in M2 macrophages and monocytes, alongside decline in CD8+ T and NK cells, in the high-risk group. This indicates that UFMylation may modulate the immune microenvironment to influence GC progression. Furthermore, we compared the drug sensitivity between the two groups. The high-risk group displayed higher sensitivity to Pazopanib, whereas the low-risk group was more sensitive to OSU-03012, Obatoclax Mesylate, NSC-87877, Mitomycin C, LY317615, GSK-650394, Gefitinib, Epothilone B, Doxorubicin, CP724714, and AS601245.

This model integrates molecules with diverse yet interconnected roles in cancer biology, spanning core tumor-promoting mechanisms to emerging regulatory functions. It encompasses key drivers of GC progression and invasion. Specifically, BVES functions as a cell adhesion molecule, and its reduced expression in GC is directly linked to tumor progression, poor survival, and the transition from intestinal metaplasia to carcinoma (27, 28). By inhibiting the ERK-NF-κB pathway the ERCAVIN2 as a tumor suppressor blocks EMT and metastasis (29). Conversely, CDH6, a type II cadherin abnormally re-activated in cancers (30, 31), contributes to invasion, migration, and mitochondrial fission in GC cells (32). COL10A1 facilitates tumor growth by activating the PI3K/Akt/mTOR and JNK/MAPK cascades (33), while MMP16 facilitates invasion by activating pro-MMP2, which degrades basement membrane components like type IV collagen to enable tumor cell migration (34–36).

Beyond these local effectors, the signature also includes genes involved in systemic signaling and microenvironment regulation. PGR, a nuclear receptor regulating proliferation and differentiation, is implicated in GC progression. Elevation of PGR correlates with LNM, altered immune cell infiltration, and unfavorable prognosis in GC (37). Besides, GDF7 is implicated in tendon repair/regeneration (38, 39), osteogenic differentiation (39–41), and immunoregulation (42). NSG2 has been determined as an immune and prognostic marker in BC and to be related to checkpoint expression, immune cell abundance, and adverse outcomes (43). In GC, APOD, involved in lipid metabolism and inflammation, correlates with adverse outcomes, altered TME, and diminished immunotherapy response (44). Similarly, LBH, via the integrin/FAK/Akt pathway, enhances invasion and proliferation of GC cells, which serves as an independent marker for adverse GC outcomes (45, 46).

Furthermore, the model captures genes exhibiting context-dependent or suppressive roles in tumorigenesis. NPTX1 demonstrates dual functions: In head-neck squamous cell carcinoma, its silencing promotes proliferation, migration, and EMT (47); in hepatocellular carcinoma, it suppresses growth and induces apoptosis via an AKT-mediated mechanism (48). ZNF662 functions as a tumor suppressor in triple-negative BC, which is decreased due to hyper-methylation of its frequent promoter, leading to inhibition of STAT3 and PI3K/AKT pathways via NGF downregulation (49). Finally, WDR17 remains understudied, with its primary expression and function reported in the eyes (50, 51), and its potential role in cancer biology awaiting further elucidation.

While the above description highlights the individual contributions of these genes, it is imperative to consider an integrated mechanistic framework that connects upstream UFMylation dysregulation with this 13-gene UARM. The direct regulatory axis between UFMylation and these specific transcripts was not experimentally established in this study, but emerging evidence suggests plausible intermediary mechanisms. UFMylation is known to modulate key oncogenic processes, including endoplasmic reticulum (ER) stress response, NF-rgisignaling, and translational control, largely through its well-characterized substrates such as the ribosomal subunit RPL26 and the ER-resident factor C53. Dysregulation of these core UFMylation pathways can create a pro-tumorigenic cellular environment that transcriptionally reprograms the GC cells. For example, persistent ER stress and activated NF-hat tranling can coordinately upregulate invasion-related genes (e.g., MMP16, CDH6) and immunomodulatory factors (e.g., APOD, PGR, NSG2), while simultaneously suppressing tumor suppressors (e.g., BVES, CAVIN2). Moreover, characterized by M2 macrophage enrichment and CD8+ T/NK cell depletion, the skewed immune microenvironment observed in the high-risk group may further reinforce the expression patterns of these signature genes through cytokine-mediated feedback loops. Within this network, GDF7 emerges as a potential critical downstream node, and its expression is possibly sustained by the UFMylation-perturbed inflammatory and regenerative signaling environment. This hypothetical model, whereby UFMylation governs a concerted transcriptional and immunological program, provides a unified explanation for the co-expression and prognostic relevance of the 13 genes, and positions GDF7 as a functionally important target within this UFMylation-driven pathogenic cascade. Future studies employing UFMylation-specific proteomics and chromatin imm noprecipitation are warranted to validate these proposed molecular links.

Conflicting results were obtained in previous studies; Some indicate that GDF7 mitigates proliferation and metastasis in hepatocellular carcinoma (52), while others suggest GDF7 variants may increase the risk of esophageal adenocarcinoma (53, 54). To clarify the impact of GDF7 on GC prognosis, GC cell lines underwent GDF7 knockdown. GDF7 knockdown obviously suppressed cell proliferation, migration, and invasion, thereby impeding GC progression. *In vivo* experiments further confirmed that GDF7 knockdown suppressed tumor growth, consistent with our findings that high GDF7 expression was linked to an adverse prognosis. This suggests the role of GDF7 as a target in GC treatment. These results enrich our cognition of the mechanism of UFMylation in GC and also present experimental evidence for developing novel targeted therapeutic strategies.

Nonetheless, several limitations are worth noting. First, data from the TCGA and GEO were utilized, so validation should be conducted using additional independent datasets. Second, direct clinical corroboration was lacking, so further investigation using clinical samples is necessary to better delineate the roles of these URGs. Third, the specific patient population most likely to benefit from this UARM-based stratification was not prospectively defined. Our analysis encompassed a heterogeneous cohort. Future studies should delineate the model’s predictive performance within clinically relevant subgroups, such as patients stratified by Lauren classification, microsatellite instability status, or those receiving immunotherapy versus standard chemotherapy. This is essential for determining whether the UARM is universally applicable or particularly informative for a specific clinical subset. Fourth, while GDF7 is proposed as a therapeutic target, its physiological functions in tendon repair, osteogenic differentiation, and potential immunoregulation raise concerns about on-target toxicity. Systemic inhibition of GDF7 may impair musculoskeletal homeostasis or wound healing. Thus, future therapeutic development must carefully evaluate these potential side effects, and strategies such as tumor-specific delivery systems, antibody-drug conjugates, or transient, conditional targeting may be required to create a favorable therapeutic window.

From a clinical translational standpoint, our findings provide a preliminary roadmap for personalized therapy for GC. The UARM can serve as a decision-support tool to guide medication: Patients classified into the high-risk group by the model exhibited elevated sensitivity to the multi-kinase inhibitor Pazopanib, suggesting that anti-angiogenic therapy may preferentially benefit this subgroup with an adverse UFMylation-associated immune microenvironment. Conversely, the low-risk group showed broad sensitivity to multiple chemotherapeutic and targeted agents, including Doxorubicin, Gefitinib, and Mitomycin C, indicating that these patients may be candidates for conventional or EGFR-targeted regimens. These predictions warrant validation in prospective clinical trials. Regarding GDF7-targeted drug development, the consistent suppression of GC cell growth and tumorigenicity upon GDF7 knockdown nominates it as a promising therapeutic target. Given the structural similarity among BMP/GDF family members, high selectivity to GDF7 should be prioritized in the future development of neutralizing antibodies or small-molecule inhibitors to avoid off-target effects on other family members with tumor-suppressive functions. Additionally, considering the immunoregulatory roles of GDF7 and the immunosuppressive TME observed in the high-risk group, combining GDF7 blockade with immune checkpoint inhibitors may represent a synergistic therapeutic strategy. These insights enhance the clinical translational value of our study, bridging UFMylation biology with actionable therapeutic interventions.

In summary, this study represented the first systematic investigation of URG expression and clinical significance, and successfully established a reliable GC prognostic model. The findings highlighted the critical function of UFMylation during GC progression and identified GDF7 as a potential target in treatment. Future research is required to clarify the molecular mechanism of UFMylation regulating GC progression, ultimately advancing precision medicine and ameliorating outcomes in GC.

## 5 Conclusion

A UARM for GC was developed and validated. This model served as a reliable and independent prognostic biomarker, which effectively stratified patients into two risk groups. Furthermore, the UARM is associated with specific immune microenvironments and drug sensitivity, offering insights for personalized GC treatment. The functional role of a core gene, GDF7, in promoting tumor progression was also experimentally confirmed.

## Data Availability

The original contributions presented in the study are included in the article/**Supplementary Material**. Further inquiries can be directed to the corresponding authors.

## Supplementary material

Supplementary Figure 1 Raw data of Western blotting.
