# Supplementary material for: An integrative analysis of UFMylation-related genes reveals a prognostic signature and identifies GDF7 as a novel therapeutic target in gastric cancer
Source: Front Oncol. 2026 Jul 27;16:1853513. doi: 10.3389/fonc.2026.1853513 (PMC13453740; doi:10.3389/fonc.2026.1853513)
Supplement: Supplementary file 1 [file DataSheet1.docx]

**Supplementary Materials**

**Supplementary Figure S1** Raw data of Western blotting.
